# Mental Health Evaluation of Younger and Older Adolescents Referred to the Center of Expertise on Gender Dysphoria in Amsterdam, The Netherlands

**DOI:** 10.1007/s10508-024-02940-3

**Published:** 2024-07-09

**Authors:** Frédérique B. B. de Rooy, Marijn Arnoldussen, Anna I. R. van der Miesen, Thomas D. Steensma, Baudewijntje P. C. Kreukels, Arne Popma, Annelou L. C. de Vries

**Affiliations:** 1 Department of Child and Adolescent Psychiatry, Amsterdam UMC, Amsterdam, The Netherlands; 2 Center of Expertise on Gender Dysphoria, De Boelelaan 1117, PO Box 7057, 1007 MB Amsterdam, The Netherlands; 3 Department of Medical Psychology, Amsterdam UMC, Amsterdam, The Netherlands

**Keywords:** Gender incongruence, Transgender, Adolescence, Mental health, Autistic traits, Gender dysphoria, DSM-5

## Abstract

The present study aimed to investigate whether differences exist between younger and older presenting adolescents at the Center of Expertise on Gender Dysphoria regarding psychological functioning and autistic traits. A total of 1487 consecutively assessed adolescents between 2000 and 2018 were divided in younger presenters (age ≤ 13.9 years) and older presenters (age ≥ 14 years). Of younger presenters, 227 (41.1%) were assigned male at birth and 325 (58.9%) assigned female at birth. In older presenters, 279 (29.8%) were assigned male at birth and 656 (70.2%) assigned female at birth. Behavioral and emotional problems were assessed with the Child Behavior Checklist (CBCL) and the Youth Self-Report (YSR). For autism traits, the Social Responsiveness Scale (SRS) was used. Compared to younger presenters, on both the CBCL and YSR older presenters had higher Total Problem (*β* = 1.75, *p* = .005, CI 0.53–2.97, *R*^2^ = .04 and *β* = 4.20,* p* < .001, CI 2.99–5.40, *R*^2^ = .07, respectively) and Internalizing Problem (*β* = 4.43, *p* < .001, CI 3.13–5.74, *R*^2^ = .06 and *β* = 6.69, *p* < .001, CI 5.31–8.07, *R*^2^ = .12, respectively) scores. Regarding autistic traits, a higher mean SRS total score was found in older presenting assigned males at birth (*β* = 4.55, *p* = .036, CI 0.30–8.81, *R*^2^ = .34). In assigned females at birth, no statistically significant difference between older and younger presenters was found in mean SRS total score (*β* = 1.19, *p* = .063, CI − 0.07 to 2.45, *R*^2^ = .39). Differences in mental health exist between younger and older presenting adolescents and call for an individualized approach in the clinical care of transgender adolescents.

## Introduction

In gender incongruent youth, the sex assigned at birth does not align with the experienced gender identity (World Health Organization, 2019). When gender incongruence is combined with clinically significant distress, this is referred to as gender dysphoria, the diagnostic term in the 5th edition of the *Diagnostic and Statistical Manual of Mental Disorders* (DSM) (American Psychiatric Association, 2013). Gender incongruence and gender non-conformity are often accompanied by mental health difficulties in both clinically referred (de Vries et al., 2011, 2016; van der Miesen et al., 2020) and community samples (Guz et al., 2021; van der Miesen et al., 2018d). Related, increased suicidality among transgender youth has been reported (Atteberry-Ash et al., 2021; Chen et al., 2021; de Graaf et al., 2022).^1^

Up until recently, studies on the mental health of clinically referred gender incongruent youth approached all adolescents as one age group. Especially samples in early follow-up studies of gender-affirming medical treatment, including puberty suppression, consisted of young people who fulfilled strict eligibility criteria. For example, adolescents were only eligible for treatment when there was a history of childhood gender incongruence and an absence of interfering mental health difficulties, such as acute suicidality (de Vries et al., 2011, 2014).

However, more recent research on adolescent referrals showed that adolescents currently presenting to gender identity services may consist of a more heterogeneous group. They show greater diversity in their gender identity, such as nonbinary identities, and sometimes experience more co-occurring psychiatric problems compared to youth in earlier studies (de Graaf & Carmichael, 2019; Kaltiala-Heino et al., 2015; Sevlever & Meyer-Bahlburg, 2019). Some also suggest that in recent years new developmental pathways have emerged, where external factors and/or mental health difficulties play a role in the onset and presentation of gender incongruence (Littman, 2019). Moreover, demographic changes have been observed in adolescent referrals to gender clinics, with recent referrals disproportionally consisting of those who were assigned female at birth (Aitken et al., 2015; Arnoldussen et al., 2020).

Studies on the timing of presentation to gender identity services (i.e., the first time a young person attends a gender identity service) have shown that presenting at an older age in adolescence is associated with worse mental health (Chen et al., 2021; Sorbara et al., 2020). Differences between “younger presenting” and “older presenting” adolescents were found as well in an earlier study at the Center of Expertise on Gender Dysphoria (CEGD) on the age distribution of adolescents assessed between 2000 and 2018. This study showed that adolescents tended to attend our gender clinic for the first time either around age 11/12 (median age 11.9 years) or around the age of 16/17 (median age 16.3 years). This finding confirmed that there is a “younger presenting” group and an “older presenting” group, with a cutoff age of 14 years based on data observation. Of interest, the older presenting group was larger than the younger presenting group and the older presenting group had a larger percentage of assigned females at birth, making up 70% of the total in this age group compared to 58.9% in the younger presenting group. Apart from demographic differences between the groups, younger presenters more often started with gender-affirming medical treatment, showed more gender nonconformity in childhood, and generally had a better body image, suggesting there may be different developmental pathways leading adolescents to attend a gender identity service (Arnoldussen et al., 2023).

In previous studies, age is seen to play a role in the development of mental health difficulties. Adolescents who presented to gender clinics either at an older age or in a more advanced stage of puberty were found to have more mental health difficulties than youth who presented at a younger age (Chen et al., 2021; Holt et al., 2016; Kaltiala-Heino et al., 2015; Sorbara et al., 2020). Another earlier study, published at a time that early medical intervention was usually not considered, already showed that gender clinic referred children below age 13 had less behavioral and emotional problems compared to adolescents age 13 and older (Zucker et al., 2002).

Not just age, but also sex assigned at birth seems to affect the occurrence of mental health difficulties in gender-referred youth, with those assigned female at birth often being more prone to internalizing problems, such as symptoms of depression, anxiety, and suicidality (Atteberry-Ash et al., 2021; de Graaf et al., 2022; Holt et al., 2016; Kuper et al., 2020).

Lack of social support, access to care, and recognizing gender incongruence at an older age were found to be related to presenting to gender identity services at an older age and may contribute to experiencing mental health difficulties as well (Chen et al., 2021; Sorbara et al., 2021). From previous research performed in our center and internationally, it is known that peer relations influence psychological functioning of gender-referred youth (de Vries et al., 2016).

A group that requires specific attention when it comes to mental health are adolescents with gender incongruence and co-occurring autism spectrum disorder (ASD). The co-occurrence of ASD in gender incongruent children and adolescents has been widely reported (de Vries et al., 2010; Holt et al., 2016; Leef et al., 2019; Mahfouda et al., 2019; Strang et al., 2018b; van der Miesen et al., 2018b; Warrier et al., 2020), and both ASD and gender incongruence are associated with experiencing additional mental health difficulties. Although little research has been done to assess the psychological functioning of adolescents with intersecting gender incongruence and ASD, the available studies show that these adolescents seem especially at risk for mental health problems (Mahfouda et al., 2019; van der Miesen et al., 2018c). While studies show a higher occurrence of ASD across the whole age range of gender incongruent youth, no studies have differentiated within the group of adolescents or taken the possible influence of age at presentation on mental health into consideration. However, a previous study in the Amsterdam adolescent cohort showed that adolescents with gender incongruence and ASD presented themselves to the clinic at a relatively older age (de Vries et al., 2010).

One important reason for the increased vulnerability of mental health difficulties in gender incongruent adolescents is related to an increase in distress of the undesired physical changes transgender youth experience when puberty starts (Chen et al., 2021; Fisher et al., 2017; Kuper et al., 2020; Steensma et al., 2013). In that case, medical gender-affirming treatment that helps to align the body with the experienced gender can also aid to alleviate mental health symptoms. Several outcome studies of medical gender-affirming treatment show improvements in body satisfaction and/or psychological functioning (Achille et al., 2020; Costa et al., 2015; de Vries et al., 2011; Kuper et al., 2020), although recent systematic reviews assess these outcomes as low-quality evidence (Ludvigsson et al., 2023; Taylor et al., 2024a, 2024b). However, none of these studies take possible differences within the adolescent population into account.

Knowledge on the association between age of presentation and mental health could prepare clinicians better for what they might expect and need for the care of gender clinic referred adolescents. While some studies conclude that early medical intervention to possibly prevent suffering should be aimed at for all transgender adolescents (Arnoldussen et al., 2020; Chen et al., 2021; Kuper et al., 2020), one could also conclude that gender identity services are dealing with a heterogeneous group of youth and that there is no “one size fits all” approach. This line of thinking has gained more attention in research, with two recent studies applying a more individualized and differential approach, one in understanding mental health problems and the other in treatment approach (Atteberry-Ash et al., 2021; Nieder et al., 2021).

In summary, it is key to better understand gender incongruent youth and the different ways in which they present to gender identity services. A recent study by Arnoldussen et al. (2023) in our clinic identified differences between younger and older presenting adolescents assessed at the CEGD regarding demographic and gender-related characteristics. The current study aims to build upon the findings of Arnoldussen et al., using the same sample and procedure, and investigates whether differences also exist in the occurrence of emotional and behavioral problems, as well as autistic traits, between younger and older presenters using a multi-informant approach. Based on the existing literature and our own clinical observations, we expect to find more mental health difficulties in the older presenting group.

The information from the present study will aid in further developing our care to better suit the needs of the diverse population that is seen in gender identity services today and offer more individualized and tailored transgender care.

## Method

### Participants and Procedure

**Fig. 1:**
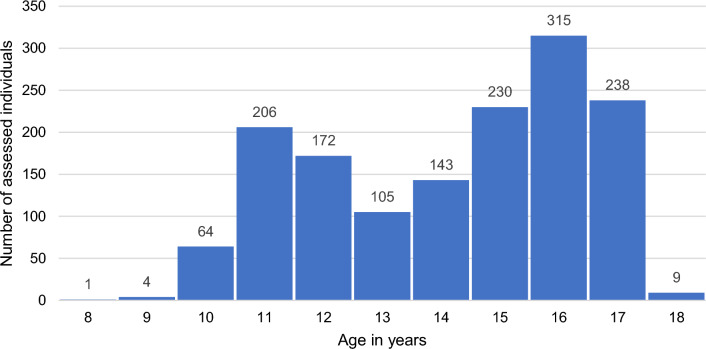
Number of assessed individuals by age in years

The sample in this study consisted of all 1,487 consecutive referrals who were assessed (age range, 8.9–18.4 years) at the CEGD between 2000 and 2018. At the CEGD clinic, the term “adolescents” is used for all consecutively assessed youth below 18 years who have started puberty or are very close to starting puberty and for whom puberty blocking interventions might be considered. These youth could be as young as 8.9 years, as puberty may start at that age. At our clinic, the start of puberty is determined by our center’s pediatricians. The pediatrician’s examination may reveal very early physical signs of the start of puberty from which they expect that they will progress to Tanner stage 2 in a short amount of time. If, however, a 9 or 10 year old presents with no signs of puberty yet, and there is no request to discuss medical interventions, the child protocol instead of the adolescent protocol is used at the CEGD (for a description of the child and adolescent protocol, see de Vries & Cohen-Kettenis, 2012). For an overview of the number of individuals per age group in the current study population, see Fig. 1.

As part of the standard procedure in the diagnostic phase at the CEGD, sociodemographic characteristics and various questionnaires on general and psychological functioning were collected upon first assessment.

In accordance with the recent Arnoldussen et al. (2023) study on age distribution of our adolescent cohort at the CEGD, the current study sample was split into “younger presenters” and “older presenters.” Arnoldussen et al. (2023) performed a median split based on data observation of a stem-and-leaf plot and a histogram, with adolescents 13.9 years or younger at first assessment being considered “younger presenters” (*n* = 552) and adolescents 14.0 years or older at first assessment “older presenters” (*n* = 935). Further details of this procedure are provided in Arnoldussen et al. (2023).

### Measures

#### Demographic and Clinical Characteristics

Demographic measures and clinical characteristics such as age at assessment, sex assigned at birth, gender dysphoria diagnosis according to the DSM (IV-TR or 5, depending on the year of assessment (American Psychiatric Association, 2000, 2013), whether or not someone started with gender-related medical treatment (“treatment status”), parents’ marital status, parents’ educational level, and Full-Scale IQ (FSIQ) were collected. Similar to previous studies at the CEGD, FSIQ was measured with the Dutch version of the Wechsler Intelligence Scale for Children or the Wechsler Adult Intelligence Scale, depending on the age of the adolescent (Wechsler, 1991, 1997, 2003). A more detailed description of these variables and how they were coded is provided in Arnoldussen et al. (2023).

#### Behavioral and Emotional Problems

Dutch versions of the Child Behavior Checklist (CBCL) (Achenbach & Edelbrock, 1983; Verhulst et al., 1996) and the Youth Self-Report (YSR) (Achenbach & Edelbrock, 1987; Verhulst et al., 1997) were used to assess both parent- and self-reported emotional and behavioral problems in our study population. The CBCL and YSR provide a total problem score, a score for internalizing problems such as anxiety and depression, and a score for externalizing problems such as aggressive or oppositional behavior.

The CBCL and the YSR are widely used to measure emotional and behavioral problems and have high internal consistency (*α* = 0.95 and *α* = 0.92, respectively). The CBCL consists of a total of 118 items, rated on a 3-point scale: “not true,” “somewhat or sometimes true,” and “very true or often true” asking parents or caregivers about emotional and behavioral problems during the past 6 months. The YSR is comprised of 102 items. Each item is rated on a 3-point scale for the past 6 months: “not true,” “somewhat or sometimes true,” or “very true or often true.”

For both the CBCL and the YSR, the Total Problem *T* score, the Internalizing problems *T* score, the Externalizing problems *T* score*,* and the percentage of scores falling in the clinical range (> 90th percentile in non-referred samples) for these outcomes were determined. The *T* scores were calculated based on Dutch norm scores (Achenbach and Edelbrock, 1983, 1987; Verhulst et al., 1996, 1996, 1997).

#### Suicidal Behavior and Peer Relations

From the CBCL and YSR, two other scales were coded: suicidal behavior and peer relations. To measure suicidal behavior, the mean of the sum of Items 18 and 91 from the CBCL and YSR was used (Van Meter et al., 2018). Item 18 reads as “Deliberately harms self or attempts suicide” (CBCL) or “I deliberately try to hurt of kill myself” (YSR) and Item 91 as “Talks about killing self” (CBCL) or “I think about killing myself” (YSR). Scores can range from 0 to 2, with a higher score indicating a higher degree of suicidality. Three Items on the CBCL and YSR contain information about peer relations (Item 25: “Does not get along with other kids” or “I don’t get along with other children,” Item 38: “Gets teased a lot” or “I get teased a lot,” and Item 48: “Not liked by other kids” or “I am not liked by other children”). From the means of these Items, scales measuring peer relations were created. Scores can range from 0 to 2, with a higher score indicating poorer peer relations (Zucker et al., 1997). All Cronbach’s α for the suicidality and peer relations scales were between 0.59 and 0.71.

#### Autistic Traits

In 2013, a Dutch version of the Social Responsiveness Scale (SRS) became available and has since been used as a screening tool in our clinic to identify the presence and extent of features typically associated with autism (Constantino et al., 2003; Roeyers et al., 2015). The SRS has good reliability, validity, and internal consistency (*α* = 0.81). It consists of 65 items, rated on a 4-point Likert scale from “not true” to “almost always.” The Dutch version of the SRS was used in this study and was completed by parents or caregivers. Total *T* scores were calculated based on the Dutch norm scores (Roeyers et al., 2015). A total *T* score from 61 to 75 indicates a mild to moderate presence of autism traits and a total *T* score of ≥ 76 a large presence of autism traits and is considered the clinical screening cutoff score suggestive of an ASD diagnosis (Constantino et al., 2003; Roeyers et al., 2015).

### Statistical Analyses

First, independent *t*-tests and chi-square tests were used to examine differences in demographic characteristics of the younger presenting and the older presenting group. Second, independent *t*-tests and zero order correlations were used to identify demographic and clinical variables potentially associated with the main outcome variables (CBCL total, internalizing, externalizing, and suicidality scale; YSR total, internalizing, externalizing, and suicidality; and SRS), clarifying which to include as control variables in the further analyses.

Assigned sex at birth, parents’ marital status, and treatment status were identified as covariates and were thus added to all regression analyses. Additional regression analyses were performed for the CBCL Total Problem and YSR Total Problem scales with the CBCL peer relations scale added to the covariates described above. In addition, as increased emotional and behavioral problems based on the CBCL total *T* score was found to be significantly correlated with the SRS, this variable was controlled for subsequently in the SRS analyses.

Third, linear and logistic regression analyses were performed to compare younger presenters and older presenters regarding outcomes on the CBCL, YSR, and SRS on total score and clinical range scores.

Finally, effect modification of assigned sex at birth and treatment status was examined. *p* values of < 0.05 were considered statistically significant. Statistical Package for the Social Sciences 26 (SPSS Inc., Chicago, IL, USA) was used to perform all statistical analyses.

## Results

### Demographic and Clinical Characteristics

Demographic characteristics of younger and older presenters are shown in Table 1. The sample consisted of significantly more AFAB than AMAB, with 58.9% of younger presenters and 70.2% of older presenters AFAB. Moreover, significantly more younger than older presenters lived with both biological parents, were diagnosed with gender dysphoria, and started with medical treatment. No significant differences between younger and older presenters were found in parents’ educational level and FSIQ.

**Table 1:** Demographic and clinical characteristics of younger presenters and older presenters

Baseline characteristic	Younger presenters *n* = 552	Older presenters *n* = 935	
Age at assessment in years, median (IQR)	11.9 (1.3)	16.3 (1.6)	
Age range in years	8.9–13.9	14.0–18.4	
Birth-assigned gender, *n* (%)			
Assigned males at birth	227 (41.1%)	279 (29.8%)	*χ*^2^(1, *n* = 1,487) = 19.69, *p* < . 001
Assigned females at birth	325 (58.9%)	656 (70.2%)	
Parents’ marital status, *n* (%)			
Living with both biological parents	352 (63.8%)	462 (49.4%)	*χ*^2^(1, *n* = 1,427) = 24.78, *p* < .001
Other	186 (33.7%)	427 (45.7%)	
Unknown	14 (2.5%)	46 (4.9%)	
Parents’ educational level, *n* (%)			
Vocational educated	251 (45.5%)	363 (38.8%)	*χ*^2^(1, *n* = 1,357) = 2.76, *p* = .097
Higher vocational or academic educated	271 (49.1%)	472 (50.5%)	
Unknown	30 (5.4%)	100 (10.7%)	
Full-scale IQ, mean (SD)	99.53 (15.32)	99.33 (16.21)	*t*(1262) = 0.217, *p* = .828
Diagnosis, *n* (%)			
Gender dysphoria diagnosis	490 (88.8%)	754 (80.6%)	*χ*^2^(1, *n* = 1,404) = 4.60, *p* = .032
No gender dysphoria diagnosis	49 (8.9%)	111 (11.9%)	
Unknown	13 (2.4%)	70 (7.5%)	
Treatment status			
Started with gender-related medical treatment	470 (85.1%)	683 (73.0%)	*χ*^2^(1, *n* = 1,487) = 29.16, *p* < .001
Did not start with gender-related medical treatment	82 (14.9%)	252 (26.9%)	

The “Unknown” option was not included in the chi-square analyses for the variables parents’ marital status, parents’ educational level and diagnosis

IQR—interquartile range, SD—standard deviation

### Behavioral and Emotional Problems

Table 2 shows the CBCL and YSR scores for younger and older presenters and the percentage of adolescents with scores in the clinical range. No significant interaction effects with age group and assigned sex at birth were found on any of the CBCL and YSR metrics. An overview of all regression analyses is presented in Table 3. The regression analyses showed that older presenters had significantly higher *T* scores on the CBCL Total problem scale and Internalizing problems scale when controlling for assigned sex at birth, parents’ marital status, and treatment status (*β* = 1.75, *p* = 0.005, CI 0.53–2.97, *R*^2^ = 0.04 and *β* = 4.43, *p* < 0.001, CI 3.16–5.74, *R*^2^ = 0.06 respectively). No significant differences between younger and older presenters were found on the CBCL Externalizing problems scale (*β* = 0.60, *p* = 0.387, CI − 0.76 to 1.97, *R*^2^ = 0.02). Adding the CBCL peer relations scale to the CBCL Total problem scale regression model led to a decrease in *β* from 1.75 to 0.97, remaining marginally significant (*β* = 0.97, *p* = 0.071, CI − 0.08 to 2.01, *R*^2^ = 0.30), indicating that CBCL total problem *T* scores are influenced by the peer relations scale, but remain higher in older presenters compared to younger presenters.

**Table 2:** Ratings of emotional and behavioral problems and percentage of adolescents with clinical range scores for the three indices on the Child Behavior Checklist (CBCL) and the Youth Self-Report (YSR) in younger and older presenters

Measure		Younger presenters	Older presenters
CBCL Total Problem *T* score	*n*	450	716
	*M*	58.96	61.23
	SD	10.53	9.94
	95% CI [LL, UL]	[57.99, 59.94]	[60.50, 61.96]
CBCL Total Problem clinical range	%	36.0	47.1
CBCL Internalizing *T* score	*n*	454	722
	*M*	59.00	63.85
	SD	11.25	10.65
	95% CI [LL, UL]	[57.97, 60.04]	[63.07, 64.62]
CBCL Internalizing clinical range	%	35.9	56.0
CBCL Externalizing *T* score	*n*	450	722
	*M*	53.51	54.58
	SD	11.77	11.02
	95% CI [LL, UL]	[52.42, 54.60]	[53.78, 55.39]
CBCL Externalizing clinical range	%	22.7	21.3
CBCL Suicidality scale	*n*	455	725
	*M*	0.10	0.26
	SD	0.27	0.45
	95% CI [LL, UL]	[0.08, 0.13]	[0.23, 0.29]
CBCL Peer Relation scale	*n*	454	725
	*M*	0.32	0.39
	SD	0.43	0.47
	95% CI [LL, UL]	[0.28, 0.36]	[0.35, 0.42]
YSR Total Problem *T* score	*n*	393	778
	*M*	54.12	58.51
	SD	9.73	9.68
	95% CI [LL, UL]	[53.15, 55.08]	[57.83, 59.19]
YSR Total Problem clinical range	%	17.6	33.2
YSR Internalizing *T* score	*n*	393	778
	*M*	54.15	60.54
	SD	10.81	11.38
	95% CI [LL, UL]	[53.08, 55.22]	[59.74, 61.34]
YSR Internalizing clinical range	%	18.8	41.1
YSR Externalizing *T* score	*n*	393	778
	*M*	49.32	51.46
	SD	9.31	9.44
	95% CI [LL, UL]	[48.39, 50.24]	[50.80, 52.13]
YSR Externalizing clinical range	%	7.9	9.9
YSR Suicidality scale	*n*	394	776
	*M*	0.11	0.34
	SD	0.32	0.50
	95% CI [LL, UL]	[0.08, 0.15]	[0.30, 0.37]
YSR Peer Relation scale	*n*	394	774
	*M*	0.30	0.40
	SD	0.39	0.44
	95% CI [LL, UL]	[0.26, 0.34]	[0.37, 0.43]

*M*—mean, SD—standard deviation, 95% CI—95% confidence interval; LL and UL indicate the lower and upper limits of the confidence interval, respectively

**Table 3:** Regression results for Child Behavior Checklist (CBCL), Youth Self-Report (YSR), and Social Responsiveness Scale (SRS) metrics using being a younger or older presenter as a criterion

Outcome measure	*β*	*p*	95% Confidence interval		*R*^2^
			LL	UL	
CBCL Total *T* score	1.75	.005	.53	2.97	.04
CBCL Internalizing *T* score	4.43	< .001	3.16	5.74	.06
CBCL Externalizing *T* score	.60	.387	− 0.76	1.97	.02
CBCL Suicidality scale	.14	< .001	.10	.19	.05
YSR Total *T* score	4.20	< .001	2.99	5.40	.07
YSR Internalizing *T* score	6.69	< .001	5.31	8.07	.12
YSR Externalizing *T* score	1.46	.015	.28	2.63	.03
YSR Suicidality scale	.21	< .001	.15	.26	.07
SRS *T* score AMAB	4.55	.036	.30	8.81	.34
SRS *T* score AFAB	1.19	.063	− 0.07	2.45	.39

The demographic covariates included in the regression models were assigned sex at birth, parents’ marital status, and whether or not a person had started medical treatment. For full regression results, see Appendix table

LL and UL indicate the lower and upper limits of the confidence interval, respectively

AMAB—assigned male at birth, AFAB—assigned female at birth

Binary logistic regressions with the same independent variables (assigned sex at birth, parents’ marital status, and treatment status) were performed for the percentages of CBCL scores within the clinical range and confirmed the results above on all scales.

For the YSR total problem *T* score, older presenters had significantly higher scores when controlling for assigned sex at birth, parents’ marital status, and treatment status (*β* = 4.20, *p* < 0.001, CI 2.99–5.40, *R*^2^ = 0.07). Adding the peer relations scale to this model gave a decrease in β of group (older vs younger presenters) from 4.20 to 3.59, remaining significant (*β* = 3.59, *p* < 0.001, CI 2.37–4.81, *R*^2^ = 0.16). Significantly higher scores for older presenters were found as well on the YSR Internalizing and Externalizing problem scales when controlling for assigned sex at birth, parents’ marital status, and treatment status (*β* = 6.69, *p* < 0.001, CI 5.31–8.07, *R*^2^ = 0.12 and *β* = 1.46, *p* = 0.015, CI 0.28–2.63, *R*^2^ = 0.03, respectively).

Similar analyses were performed for the percentages of YSR scores in the clinical range and similar results were found on the YSR Total problem and Internalizing problems scales. No significant differences between the younger and older presenting groups were found for the percentage of scores within the clinical range on the YSR Externalizing problems scale.

### Suicidal Behavior

On the suicidality scale, older presenters had a significantly higher mean score on the metric from the CBCL and the YSR when controlling for assigned sex at birth, parents’ marital status, and treatment status (*β* = 0.14, *p* < 0.001, CI 0.10–0.19, *R*^2^ = 0.05 and *β* = 0.21, *p* < 0.001, CI 0.15–0.26, *R*^2^ = 0.07, respectively).

### Autistic Traits

The Dutch version of the SRS became available in 2013 and has therefore only been completed for youth presenting after this time. In total, 501 parents/caregivers completed the SRS, of which 183 were younger presenters and 318 older presenters. As significant interaction effects with age group and assigned sex at birth were found on the SRS, these analyses were stratified by assigned sex at birth (*β* = − 4.25, *p* = 0.010, CI − 7.46 to − 1.04, *R*^2^ = 0.43). Mean total *T* scores and the percentages of scores suggestive of moderate autistic traits (from 61 to 75) or highly suggestive of an autism diagnosis (≥ 76) are shown in Table 4. In AMAB, the SRS total *T* score was significantly higher in older presenters compared to younger presenters when controlling for parents’ marital status, treatment status, and total *T* score on the CBCL (*β* = 4.55, *p* = 0.036, CI 0.30–8.81, *R*^2^ = 0.34). In AFAB, no significant difference was found between older and younger presenters in SRS total *T* score when controlling for parents’ marital status, treatment status, and total *T* score on the CBCL (*β* = 1.19, *p* = 0.063, CI − 0.07 to 2.45, *R*^2^ = 0.39).

**Table 4:** Mean total *T* scores and percentages of *T* scores 61–75 and ≥ 76 on the Social Responsiveness Scale in younger and older presenters

Measure		Younger presenters		Older presenters	
		AMAB	AFAB	AMAB	AFAB
	*n*	56	127	71	247
Total *T* score	*M*	52.89	62.49	58.93	64.72
	SD	10.03	6.57	13.41	6.61
	95% CI [LL, UL]	[50.21, 55.58]	[61.34, 63.64]	[55.75, 62.10]	[63.90, 65.55]
Percentage with *T* score 61 to 75	%	14.3	45.7	28.2	61.9
Percentage with *T* score ≥ 76	%	5.4	6.3	12.7	8.1

AMAB—assigned male at birth, AFAB—assigned female at birth, *M*—mean, SD—standard deviation, 95% CI—95% confidence interval

LL and UL indicate the lower and upper limits of the confidence interval, respectively

In AMAB, 14.3% of younger presenters and 28.2% of older presenters had a SRS total *T* score between 61 and 75. In AFAB, 45.7% of younger presenters and 61.9% of older presenters had a total *T* score between 61 to 75. Using the clinical screening cutoff score suggestive of an ASD diagnosis, 5.4% of younger presenting AMAB and 12.7% of older presenting AMAB had total *T* scores ≥ 76, while in AFAB, 6.3% of younger presenters and 8.1% of older presenters had a total *T* score ≥ 76.

Both in AMAB and AFAB, binary logistic regression showed significant differences between younger and older presenters when controlling for parents’ marital status, treatment status, and total *T* score on the CBCL for having a total *T* score ≥ 61 (OR 2.89, *p* = 0.048, CI 1.01–8.08 in assigned males and OR 2.14, *p* = 0.010, CI 1.20–3.84 in assigned females).

### Sensitivity Analyses

To explore the influence of age, the analyses were repeated without including individuals under the age of 11. The results from these analyses were consistent with the analyses of the cohort of consecutive referrals as a whole in terms of both descriptive and inferential statistics across all metrics (results are available from the corresponding author upon request).

## Discussion

The aim of this study was to examine if mental health differences exist between younger and older presenting adolescents attending our gender identity service in Amsterdam. Overall, we found that younger presenters had fewer psychological problems and autistic traits compared to older presenters.

In both age groups, the reported problems were primarily of an internalizing nature, such as mood and anxiety, which is in line with findings of earlier studies (Arnoldussen et al., 2023; Atteberry-Ash et al., 2021; de Vries et al., 2011; Holt et al., 2016; Kuper et al., 2019; van der Miesen et al., 2020).

In addition, suicidal thoughts and behavior were more often reported in older presenters than in younger presenters. All of these results were found on both the parent- and self-report measures. Moreover, older presenting adolescents reported more autistic traits compared to younger presenters. This was most frequently seen in older presenting AFAB, with 70% of older presenting AFAB reporting a moderate or large presence of autism characteristics. About 10% of adolescents in our sample had scores suggestive of a clinical ASD diagnosis, which is in line with results of previous studies in our clinic (de Vries et al., 2010; van der Miesen et al., 2018b) and other gender identity services (Holt et al., 2016; Mahfouda et al., 2019). Scores indicating a large presence of autism traits suggestive of an autism spectrum diagnosis were seen most in older presenting AMAB.

Similar to the results in our study, differences in mental health problems and rates of suicidality between older and younger presenters were reported before by Sorbara et al. (2020) and Chen et al. (2021). While in general mental health conditions become more prevalent in late adolescence (Solmi et al., 2022), in gender incongruent youth puberty might play a role in particular. Having to experience puberty and the associated physical changes that are not in line with one’s experienced gender identity is deemed to be highly stressful and is associated with psychological problems (Chen et al., 2021; Fisher et al., 2017; Kuper et al., 2020; Steensma et al., 2013). In their study, Sorbara et al. (2020) indeed found that adolescents in a more advanced stage of puberty at intake had a higher odds of having mental health difficulties. However, puberty stage did not fully explain the higher level of mental health difficulties in older adolescents, suggesting other factors play a role as well. Community-based studies show that gender non-conforming youth experience more mental health difficulties compared to their cisgender peers (Guz et al., 2021; van der Miesen et al., 2018d), a vulnerability that is already present in childhood and remains at a similar level throughout adolescence (Fish et al., 2023).

Apart from incongruence between physical sex characteristics and gender identity, the experience of minority stress and the lack of family and/or peer support are related to mental health in young transgender persons (Atteberry-Ash et al., 2021; de Vries et al., 2016; Sorbara et al., 2021). Growing up in an unaccepting environment may be related to psychological problems in itself, but could also be related to a delay in the declaration of gender incongruence. This could reinforce distress, low mood, or other problems. In a survey study among referred transgender adolescents, the lack of family support was considered an important factor in developing mental health issues and was also of influence on the age of declaration of gender identity as reported by youth and their caregivers (Sorbara et al., 2021). In the current study, the differences in mental health outcomes between younger and older presenters were not associated with parents’ marital status and while we found that peer relations were associated with mental health outcomes, poorer psychological functioning in older presenters could not be fully attributed to peer relations.

So apart from bodily distress and social acceptance, there might also be other factors related to a later presentation of adolescents with gender issues to gender identity services. An important one may be that not all adolescents seem to be aware of, or are able to openly express, their feelings of gender incongruence at a young age. While one study found that there were no significant differences in the amount of time younger and older presenters waited to come out and present to gender identity services after recognizing their gender incongruence, they did find that older presenters were older when they first recognized their gender incongruence (Sorbara et al., 2020). A recent study at the CEGD found as well that older presenters had less recalled gender nonconformity in childhood compared to younger presenters. Moreover, older presenters were less likely to pursue gender medical affirming treatment (73%), with 85.1% doing so in younger presenters (Arnoldussen et al., 2023). These findings could suggest that older presenters possibly have different developmental pathways, exploratory trajectories, and treatment wishes compared to younger presenters.

In addition to later gender recognition, another explanation for why some adolescents present to gender identity services at an older age is that mental health difficulties may be present before a young person recognizes their gender identity issues, as is described in clinical and parent-reported studies (de Graaf & Carmichael, 2019; de Vries et al., 2010; Kaltiala-Heino et al., 2015; Littman, 2019; Sevlever & Meyer-Bahlburg, 2019). It could be that these mental health issues make exploratory trajectories of gender identities more complex and possibly result in a later presentation to gender identity services. Some suggest that the use of the internet or peer influence may play a role and that vulnerable adolescents may (mistakenly) interpret that their non-specific emotional difficulties stem from gender incongruent feelings (Kaltiala-Heino et al., 2015; Littman, 2019). The fact that older presenters were less likely to fulfill the criteria for the gender dysphoria diagnosis and to start with medical gender affirming treatment after their initial assessment may indicate that this may be the case for some of the older presenters.

A specific group of adolescents who might have a more complex exploratory trajectory and psychological assessment are those with co-occurring gender incongruence and autism (Strang et al., 2018b). Like other studies in transgender populations, we observed a high occurrence of autistic traits in our complete sample (de Vries et al., 2010; Holt et al., 2016; Kaltiala-Heino et al., 2015; Mahfouda et al., 2019; Strang et al., 2018b; van der Miesen et al., 2018b; Warrier et al., 2020), and we found differences between the younger and older presenting group. Although a high percentage of older presenting AFAB reported moderate signs of autistic traits, older presenting AMAB were more likely to meet the clinical cutoff of an ASD diagnosis. These differences between AFAB and AMAB could be related to AFAB being less likely to receive an ASD diagnosis in general (Atteberry-Ash et al., 2021); however, it could also be that the presently available measures of autistic traits are not able to adequately capture young transgender persons’ experiences (Strang et al., 2020). It has been argued that experiencing gender incongruence, and minority stress that stems from it, may in itself cause difficulties in social interactions and that the elevated scores on measures represent these social difficulties, not actual autistic traits (Holt et al., 2016; Turban & van Schalkwyk, 2018). On the other hand, studies have shown that in populations of gender incongruent youth with autistic traits all subdomains, not only the social subdomains, of the ASD spectrum are elevated, making the hypothesis that the measured autistic characteristics are solely due to minority stress from gender incongruence unlikely (Strang et al., 2018a; van der Miesen et al., 2018a). To our knowledge, the high frequency of moderate autism characteristics in transgender adolescents, especially in older presenting AFAB, has not been reported before. These findings call for further research into how the difference between assigned males at birth and assigned females at birth, a possible relation of autism traits, and the development of gender incongruence should be interpreted. Future research should also examine whether differences in mental health between younger and older presenters might be explained by the presence of ASD characteristics.

This study had several clinical implications. We have observed mental health concerns in adolescents presenting at our gender clinic, more so in those presenting at a later age. Older presenters might follow more diverse developmental and exploratory trajectories of gender identity and may have different treatment needs and desires that do not always fit in traditional care models. Therefore, trajectories of older adolescents, and differentiating for whom medical gender affirmative treatment is indicated, may be more challenging. Given these complexities, an individualized assessment phase, in which a mental health professional and young person work together to understand the young person’s views, needs, and desires and get insight into the possible co-occurring mental health issues, is in our view an important part of the care for gender incongruent adolescents. Such an individualized assessment has also been an important part of the so-called Dutch approach as well as current treatment guidelines (Cohen-Kettenis et al., 2011; Coleman et al., 2022). One could also consider the possible role of exploratory psychotherapy, which has recently gained more attention in the management of gender dysphoria in adolescents (Cass, 2024).

This study in younger and older adolescents at the CEGD is a first step in understanding transgender adolescents as a heterogeneous group. To further understand in which clinically relevant ways adolescents differ from each other when they attend gender services, future studies should differentiate between younger and older presenting adolescents. In addition, they should follow youth’s trajectories of gender identity development over time, evaluate treatment desires and outcomes, and the relation with co-occurring mental health issues on the development of gender identity and steps in (medical) transition. These types of future studies could be a step into understanding whether or not the differences found between younger and older presenting youth point toward different underlying developmental trajectories of gender dysphoria. In performing future research, an effort should be made to include diverse samples, with special attention to non-binary gender identities and other intersecting minority identities, as we know from the literature that these (intersecting) identities can lead to experiencing mental health challenges and difficulties in accessing care (Ciria-Barreiro et al., 2021; Poquiz et al., 2021). The current study could not study differences based on gender identity or minority identities, as this information was not collected at clinical entry for this study sample.

Future research should also provide more in-depth information on mental health issues and also include differences in psychological strengths, such as resilience factors and adaptive mechanisms, in younger and older presenters. In this study, the CBCL and YSR problem behavior scales were used to assess psychological functioning. In addition to this, future studies could include the CBCL and YSR Competence Scale scores as metrics of strength and resilience. A strength of the current study was the use of a multi-informant approach that provides a more adequate assessment of psychological functioning in adolescents than only self- or parent-report (de Vries et al., 2016). However, while the CBCL and YSR are widely used and well-validated measures of emotional and behavioral problems, they do not provide information on clinical diagnoses of mental health conditions. Similarly, the SRS is solely a screening tool for the presence and severity of autism traits. Also, it is debated whether this and other currently available measures of autistic traits are suitable for the transgender adolescent population (Strang et al., 2020).

Moreover, this study had a cross-sectional design and some relevant factors that could be related to the age of presentation, such as openness toward gender diversity, access to care, and experiencing minority stress, were not available in our sample.

### Conclusion

Differences exist in the mental health of younger and older presenting adolescents to our gender identity service in Amsterdam. Younger presenters reported less emotional and behavioral problems and lower scores on a suicidality metric. In both age groups, autistic traits were found, but these were most prevalent in older presenting assigned females at birth. While peer relations were significantly associated with mental health outcomes, the poorer psychological functioning in older presenting adolescents could not be fully explained by this, suggesting other factors play a role as well. Moreover, a larger percentage of older presenters did not receive the diagnosis of gender dysphoria or started with medical gender affirming treatment and older presenters reported less gender incongruence in childhood, suggesting possible differences in the development of gender incongruence between younger and older presenters.

The combination of the greater amount of mental health concerns and possible differences in the development and exploration of gender identity and incongruence, points toward more complex and diverse trajectories in older adolescents. Further research into the differences within transgender adolescents and the diversity in how they present to gender identity services is needed. In the meanwhile, we advise an individualized approach in the clinical care of transgender adolescents including an individualized assessment phase, combined with medical gender affirming treatment when needed.

## Data Availability

The data that support the findings of this study are not available due to local privacy policies.

## Appendix

Regression results for Child Behavior Checklist (CBCL), Youth Self-Report (YSR) and Social Responsiveness Scale (SRS) metrics

**Table:**

Measure	*β*	*p*	95% Confidence Interval		*R*^2^
			LL	UL	
CBCL Total *T* score					0.04
Sex assigned at birth	− 0.67	0.293	− 1.92	0.58	
Parents’ marital status	2.79	< 0.001	1.61	3.98	
Treatment status	2.94	< 0.001	1.45	4.43	
Group	1.75	0.005	0.53	2.97	
CBCL Internalizing *T* score					0.06
Sex assigned at birth	− 1.02	0.138	− 2.36	0.33	
Parents’ marital status	1.91	0.003	0.64	3.18	
Treatment status	2.94	< 0.001	1.34	4.54	
Group	4.43	< 0.001	3.13	5.74	
CBCL Externalizing *T* score					0.02
Sex assigned at birth	0.03	0.964	− 1.37	1.43	
Parents’ marital status	2.77	< 0.001	1.45	4.10	
Treatment status	1.90	0.025	0.24	3.57	
Group	0.60	0.387	− 0.76	1.97	
CBCL Suicidality scale					0.05
Sex assigned at birth	0.03	0.191	− 0.02	0.08	
Parents’ marital status	0.07	0.002	0.03	0.12	
Treatment status	0.05	0.125	− 0.01	0.10	
Group	0.14	< 0.001	0.10	0.19	
YSR Total *T* score					0.07
Sex assigned at birth	− 1.99	0.001	− 3.19	− 0.79	
Parents’ marital status	1.42	0.014	0.29	2.54	
Treatment status	2.60	< 0.001	1.16	4.03	
Group	4.20	< 0.001	2.99	5.40	
YSR Internalizing *T* score					0.12
Sex assigned at birth	− 4.52	< 0.001	− 5.89	− 3.15	
Parents’ marital status	0.71	0.277	− 0.57	2.00	
Treatment status	2.52	0.003	0.88	4.16	
Group	6.69	< 0.001	5.31	8.07	
YSR Externalizing *T* score					0.03
Sex assigned at birth	1.14	0.056	− 0.03	2.31	
Parents’ marital status	2.10	< 0.001	1.00	3.20	
Treatment status	1.83	0.010	0.43	3.23	
Group	1.46	0.015	0.28	2.63	
YSR Suicidality scale					0.07
Sex assigned at birth	0.01	0.702	− 0.05	0.07	
Parents’ marital status	0.03	0.337	− 0.03	0.08	
Treatment status	0.12	< 0.001	0.05	0.18	
Group	0.21	< 0.001	0.15	0.26	
SRS *T* score AMAB					0.34
Parents’ marital status	2.38	0.185	− 4.47	22.82	
Treatment status	4.95	0.078	− 0.06	10.47	
CBCL Total *T* score	0.60	< 0.001	0.38	0.81	
Group	4.55	0.036	0.30	8.81	
SRS *T* score AFAB					0.39
Parents’ marital status	1.48	0.017	0.27	2.70	
Treatment status	1.10	0.205	− 0.61	2.81	
CBCL Total *T* score	0.40	< 0.001	0.34	0.46	
Group	1.19	0.063	− 0.07	2.45	

LL and UL indicate the lower and upper limits of the confidence interval, respectively

Sex assigned at birth was coded as assigned male at birth = 0 and assigned female at birth = 1

Parents’ marital status was grouped as “living with both biological parents” (coded as 1) or “other” (coded as 2)

Treatment status refers to whether someone started with gender-related medical treatment (coded as 1) or did not start with gender-related medical treatment (coded as 2)

Group refers to belonging to the group of “younger presenters” (coded as 0) or “older presenters” (coded as 1)

## Footnotes

1 Transgender youth and gender incongruent youth are used interchangeably in this paper.
